# An immunocompetent adult patient with hepatitis and guillain-barré syndrome after cytomegalovirus infection

**DOI:** 10.1186/1743-422X-8-95

**Published:** 2011-03-04

**Authors:** Ying Ma, Juan Feng, Ying Qi, Xiao Guang Dou

**Affiliations:** 1 Department of Neurology, Shengjing Hospital of China Medical University, Shenyang 110817, PR China; 2 Virus Laboratory, Shengjing Hospital of China Medical University, Shenyang 110817, PR China; 3 Department of Infectious Disease, Shengjing Hospital Affiliated to China Medical University, Shenyang 110817, PR China

## Abstract

**Objectives:**

It is to describe an immunocompetent adult patient with hepatitis and Guillain-Barré syndrome (GBS) after Cytomegalovirus (CMV) infection. In the initial course of the diagnosis and treatment, CMV infection was ignored.

**Case report:**

A 19-year-old Chinese girl complained of fatigue with pain and numbness of the limbs, with abnormal liver function. She was diagnosed as a case of GBS based on history, clinical findings and auxiliary examinations. On day 13 of admission, her liver function was still abnormal. So CMV was recommended to examine. CMV hepatitis was diagnosed on positive serum anti-CMV IgG and IgM antibodies. The case was improved only with intravenous immunoglobulin therapy, without the use of antiviral therapy.

**Conclusions:**

This case showed an immunocompetent adult patient with hepatitis and GBS induced by CMV. The physicians should take into account multi-system involvement of severe CMV infection.

## Introduction

Cytomegalovirus (CMV) belongs to the herpes virus family, DNA virus. CMV infection is most commonly sub clinical. In the immunocompromised host, primary CMV infection, reactivation and re-infection are all associated with significant morbidity and mortality [1,2]. In the immunocompetent adult, primary CMV infection is usually asymptomatic but can result in a mononucleosis syndrome [1-3]. CMV infection in immunocompetent hosts may rarely be able to lead to severe organ specific complications. But some serious complications have been reported. Severe hepatitis is a frequent presentation [4,5]. Central nervous system disorders constituted the second most frequent manifestations of CMV infection in immunocompetent patients, mainly as meningitis, encephalitis, myelitis, nerve palsies, Guillain-Barré syndrome (GBS), et al. [2,6-9] But the cases involved the liver and central nervous system are very rare. We described the first case reported in China, of CMV induced hepatitis and GBS in an immunocompetent adult patient.

## Case report

A 19-year-old Chinese girl was admitted to local hospital complaining of fatigue with pain and numbness of the limbs after the onset of an upper respiratory tract infection 10 days ago. Laboratory test showed alanine aminotransferase (ALT) and aspartate aminotransferase (AST) had been significantly elevated, but negative hepatitis virus. And the patient had no drug history. Hepatitis was diagnosed, and the patient received corresponding therapies, but the symptoms did not improve. And the patient accompanied by limb weakness and trismus 5 days before admission to Neurology Department of our hospital.

Upon physical examination after admission: the patient's blood pressure was 120/85 mmHg, respiratory rate 16 breaths per minute, heart rate 68 beats per minute, and temperature 36.8°Celsius. No obvious skin and sclera jaundice. On neurological examination, the patient was conscious and speech fluent, and her orientation, calculation, memory, attention and understanding were normal. Bilateral mild peripheral facial nerve palsy was noted as follows: bilateral eyelash sign positive, frown poor on the right, bilateral nasolabial slightly shallow, teeth show little effort, but drum gills normal. All other cranial nerves were intact. Motor strength of the limbs was grade 3. Muscle stone of the limbs decreased. There was obviously grip pain on bilateral forearm and gastrocnemius muscles. Distal limbs were allergic to pain perception. Deep tendon reflexes on the upper limbs were diminished and absent on the lower limbs. No pathological reflexes were elicited. Finger refers to the nose was accurate.

Auxiliary examination of the first day of admission: Electromyography (ECG): Motor nerve conduction velocity of bilateral median nerve and peroneal nerve was normal, but the distal latency; evoked potential amplitude of double the median nerve and right peroneal nerve reduced. Liver, gallbladder and spleen ultrasonography were normal. Lumbar puncture: pressure measured 100 mmH_2_O, and a spinal tap yielded clear, colorless cerebrospinal fluid (CSF) with the following component levels: glucose 2.89 mmol/L, protein 0.8 g/L, and chloride 121.0 mmol/L. Gram staining, ink staining and anti-acid staining were negative. On cytospin preparations, 7 cells were collected. CSF findings indicated cells-protein separation. Arterial oxygen pressure and saturation were normal. Complete blood count and chemistry levels were normal, except for ALT and AST levels of 303.8 U/L and 106.3 U/L respectively, indicating acute or chronic liver injury. But the patient had no history of liver insufficiency, and all of hepatitis virus test were negative (including A, B, C, D and E).

The patient was diagnosed as a case of GBS based on history, clinical findings and auxiliary examinations (ECG and CSF) [10]. So intravenous immunoglobulin therapy (20 g/day for five days) and intramuscular injection of vitamin B1 and B12 were instituted. However, impaired liver function was concerned since there were no significant clinical findings of liver (negative hepatitis virus and unremarkable liver ultrasonography).

On her second hospital day, the whole body pain got slightly improved. 5 days after admission, her whole body pain significantly improved and she could walk alone (Motor strength of the limbs grade 3+). But the symptoms of the right peripheral facial paralysis were more severe than the left. Liver function examined again ALT and AST levels of 172 U/L and 69 U/L respectively.

On day 13, the patient could go downstairs (Motor strength of the limbs grade 4). Liver function examined again ALT and AST levels of 147 U/L and 87 U/L respectively, indicating liver function was still abnormal. Department of Gastroenterology and Infectious Disease were invited to consult this patient. CMV infection was concerned. Positive serum anti-CMV IgG antibody (10.4 IU/mL) and IgM antibody (59.2 IU/mL) were found serially using commercially available ELISA kit. CMV-DNA by PCR in blood and urine were negative. Thus, CMV infection combined with CMV liver injury was diagnosed prompted on the patient's liver enzyme test and positive serum anti-CMV IgG and IgM antibodies. For CMV-DNA negative, antiretroviral therapy didn't be used, only protection of liver function was administered.

By day 19, the patient gradually recovered from the limbs weakness and facial paralysis and was discharged. A month follow-up tests indicated the patient was fully recovered, showing negative CMV-DNA in blood and urine and liver function normal. On neurological examination, the patient was fully recovered clinically including facial nerve normal, muscle strength and stone normal, and deep tendon reflexes normal.

## Discussion

Manifestations of CMV infection in immunocompromised hosts have been extensively reported, but those observed in immunocompetent patients have received comparatively little attention. Clinical manifestations of CMV infection include fever, pancytopenia, hepatitis, rarely colitis, pneumonia and retinitis [1,2]. Neurological involvement by CMV (such as GBS) is usually attributed to aberrant immunological responses triggered by the presence of CMV, but not due to direct infection [11]. Intensive care management with ventilatory support and the use of intravenous immunoglobulin have significantly improved outcomes in GBS patients [12]. The improvement observed in some of the treated patients of CMV may have been related to the typically self-limiting course of the disease, so no conclusive statements regarding the use of antiviral treatment can be made from the available data in the literature [2]. We described an immunocompetent adult patient with hepatitis and GBS induced by CMV. In diagnosis and treatment of this case, we initially ignored the CMV infection. The case was improved only with intravenous immunoglobulin therapy, without the use of antiviral therapy for negative CMV-DNA. The lessons of diagnosis and treatment of this case alerted clinicians that multi-system involvement should be taken into account severe CMV infection. Early recognition, assistant examination as soon as possible and prompt treatment is critical to improve overall outcomes.

## Conclusion

This case showed an immunocompetent adult patient with hepatitis and GBS induced by CMV. The physicians should take into account multi-system involvement of severe CMV infection.

## Consent

Written informed consent was obtained from the patient for publication of this case report. A copy of the written consent is available for review by the Editor-in-Chief of this journal.

## Competing interests

The authors hereby disclose any financial or personal relationship with other people or organizations that could have inappropriately influenced this work.

## Authors' contributions

YM, JF, YQ and XGD carried out the clinical examination and treatment of the patient. YM and JF participated in the drafting of the manuscript, literature search, and coordination. All authors read and approved the final manuscript.
